# Metabolic-Immune Coupling in Urologic Cancers: Macrophage Reprogramming as a Therapeutic Nexus

**DOI:** 10.7150/ijbs.127634

**Published:** 2026-02-26

**Authors:** Wenxue Huang, Weijia Li, Wentai Shangguan, Lin Yang, Zhuohang Li, Boyuan Sun, Cunzhen Ma, Xunguo Yang, Peidan Peng, Jie Zhao, Bisheng Cheng, Peng Wu

**Affiliations:** 1 Nanfang Hospital, Southern Medical University, Guangzhou, 510000, China.; 2 Department of Surgery, Division of Urology, Beth Israel Deaconess Medical Center, Harvard Medical School,Boston, MA, USA.; 3 Department of Urology, Second Affiliated Hospital of Naval Medical University, Shanghai, 200003, China.; 4 NMPA Key Laboratory for Research and Evaluation of Drug Metabolism, Guangdong Provincial Key Laboratory of New Drug Screening, School of Pharmaceutical Sciences, Southern Medical University, Guangzhou, 510515, China.

**Keywords:** Tumor-associated macrophage, metabolic reprogramming, prostate cancer, bladder cancer, renal cell carcinoma

## Abstract

Therapeutic responsiveness in urologic cancers is gated by metabolic-immune coupling that conditions tumor-associated macrophages (TAMs). Myeloid-dominated “cold” ecosystems blunt antigen handling, phagocytosis, and trafficking, limiting the benefit of immune-checkpoint inhibitors (ICIs). This review focuses on three high-value axes that shape TAM state and niche: the lactate-pH / hypoxia-HIF-VEGF axis that enforces acidic, adenosinergic suppression and angiogenic programs; lipid rafts axis that stabilizes inhibitory hubs (e.g., PI3K-AKT/TREM2) and skews phagocytosis/antigen presentation; and ferroptosis-redox axis control that sets inflammatory versus tolerogenic set-points. The review further outlines pharmacodynamic anchors—hyperpolarized ^13^C-pyruvate MRI (k_PL_), soluble ANGPT2, and spatial NT5E/ADORA2A modules—to operationalize a bench-to-biomarker-to-bedside loop using organoid-immune co-cultures, humanized/xenograft systems, and *ex vivo* tumor slices. This framework prioritizes adaptive enrichment for glycolysis- or adenosine-high tumors, rational timing/sequencing with ICIs, and avoidance of global myelosuppression. Collectively, metabolism-informed TAM re-education offers a route to convert myeloid-dominated “cold” ecosystems into treatment-responsive states across urologic cancers.

## 1. Introduction

Urologic malignancies, including prostate cancer (PCa), bladder cancer (BCa), and renal cell carcinoma (RCC), represent major contributors to global cancer incidence and mortality^1^, ^2^. Despite the remarkable success of ICIs across multiple cancer types, response rates remain highly variable between patients in urologic cancers^3^. A substantial fraction of tumors display an immune-excluded or non-inflamed microenvironment characterized by enrichment of immunosuppressive myeloid populations; these tumors less often benefit from immunotherapy^3^. Within this landscape, tumor-associated macrophages (TAMs) constitute a dominant and highly plastic myeloid compartment with a central role in shaping immune tone and treatment responsiveness.

Macrophage states are not fixed but are continuously instructed by tumor- and microenvironment-derived cues^4^. Among these, tumor metabolic rewiring is a major force that reshapes the tumor microenvironment (TME) and, in turn, immune-cell function^5^. The Warburg effect describes the preference of cancer cells for high-rate glycolysis even in the presence of oxygen, accompanied by lactate accumulation^6^. This metabolic configuration supports proliferative demands while driving hallmark TME features, including nutrient competition, regional hypoxia, extracellular acidosis, and enrichment of metabolites such as lactate and adenosine^7^-^9^. Beyond glycolysis, dysregulated lipid and iron metabolism in tumor cells intersects with the metabolic needs of immune cells, generating a multi-layered metabolic-immune coupling that can bias TAM programs toward immunosuppression^10^, ^11^.

In this review, we posit that metabolic-immune coupling constrains therapeutic responses in urologic cancers largely by shaping TAM plasticity and niche adaptation. Urologic tumors exhibit disease-specific metabolic features and establish reciprocal metabolic interactions with immune and stromal compartments, together reinforcing an immunosuppressive milieu^12^, ^13^. Recent advances in single-cell transcriptomics, spatial profiling, and multi-omics integration have begun to resolve how TAM heterogeneity aligns with local metabolic constraints and how these constraints may be pharmacologically perturbed^14^.

Distinct from prior reviews that discuss macrophages or cancer metabolism in isolation, this review offers a TAM-centered, clinically oriented framework for urologic malignancies by organizing current evidence into three high-value metabolic axes that repeatedly converge on macrophage function and immune contexture: the lactate-pH / hypoxia-HIF-VEGF axis, the lipid rafts axis, and the ferroptosis-redox axis. We connect these axes to spatially patterned TAM states and to practical translational questions, including which niches are plausibly rate-limiting in PCa, BCa, and RCC, what macrophage phenotypes are expected to shift upon intervention, and which pharmacodynamic readouts may confirm pathway engagement in patients. By integrating mechanistic biology with models, biomarkers, and trial considerations, we aim to provide an executable roadmap for metabolism-informed TAM reprogramming and rational immunotherapy combinations in urologic cancers.

## 2. TAM origins, states, and niches in urologic cancers

### 2.1 Origins and heterogeneity of TAMs

TAMs in urologic cancers display pronounced heterogeneity, which mainly arises from dual ontogeny—embryonically derived tissue-resident macrophages maintained by local proliferation and monocyte-derived macrophages recruited from the circulation^4^, ^15^, ^16^. Traditionally, based on *in-vitro* activation paradigms, macrophages were simply divided into the classically activated, pro-inflammatory M1 type and the anti-inflammatory, pro-tumor M2 type^17^, ^18^. In subsequent studies, M2 macrophages were further subdivided into M2a, M2b, M2c, and M2d according to the stimuli^19^-^22^. However, this binary taxonomy is overly simplistic within the authentic TME and fails to capture the functional diversity of TAMs. Modern single-cell transcriptomics reveals a continuum of states in urologic tumors, including phagocytic/antigen-presenting, angiogenic, immunoregulatory, and immune-stimulatory subsets.

Studies in murine PCa models indicate that, compared with resident TAMs, monocyte-derived TAMs are more prone to immunosuppressive phenotypes and promote tumor progression^23^. However, in RCC and BCa, systematic lineage-tracing data in human tissues remain limited, and the relative ontogeny contributions are yet to be clarified.

### 2.2 Organ-specific TAM niches

Distinct metabolic and immune contexts across urologic organs sculpt unique TAM niches and create disease-specific therapeutic challenges:

**Prostate cancer.** TAMs often cluster near androgen-synthesizing glands and exhibit conspicuous lipid-loaded phenotypes. Cholesterol burden drives liver X receptor (LXR) and peroxisome proliferator-activated receptor γ (PPARγ) pathways, inducing immunoregulatory TAM states. One study suggested that lipid-rich TAMs enhance tumor progression and suppress clearance by altering membrane architecture and reducing antigen-presentation capacity^24^. Androgen signaling may affect lipid metabolism, leading to lipid loading in TAMs and thereby suppressing their phagocytic activity and antigen presentation.

**Bladder cancer.** TAMs encounter distinctive metabolic challenges, notably acidic and adenosinergic niches generated by high lactate and purine metabolism. BCa typically shows intense glycolysis with abundant lactate production, which promotes regulatory TAM states and shapes a strongly immunosuppressive TME^25^. In such environments, TAMs tend to polarize toward an M2-like phenotype; M2 cells further suppress antitumor immunity by producing IL-10 and TGF-β. Studies show that downregulation of SPOP, an E3 ubiquitin ligase, in BCa promotes cancer stemness and macrophage recruitment/polarization via the STAT3/CCL2/IL-6 axis, creating a symbiotic relationship wherein tumor cells and TAMs mutually reinforce each other^26^.

**Renal cell carcinoma.** Originating from metabolically active renal tubular epithelial cells, RCC harbors TAMs with unique lipid-droplet accumulation and hypoxia-inducible factor (HIF) dependence. Due to von Hippel-Lindau tumor-suppressor gene (VHL) mutations, lipid droplets accumulate and HIF pathways are constitutively activated, creating hypoxia that restricts antigen presentation and fosters pro-angiogenic TAM phenotypes. Against a background of constitutive HIF-α activation caused by VHL mutations, metabolic reprogramming in renal cancer cells profoundly influences TAM states^27^. In fumarate hydratase-deficient renal cell carcinoma (FH-deficient RCC), glycolytic activity is markedly increased due to the Warburg effect, and closely associated with resistance to ICIs. An integrated multi-omics analysis identified a glycolysis signature (Glyc.Sig) that inversely correlates with ICI efficacy, and pinpointed lactate dehydrogenase A (LDHA) as a potential combinatorial target^28^.

### 2.3 Spatial ecology of TAMs

Spatial ecology is increasingly recognized as a major organizer of TAM heterogeneity in urologic cancers. Spatial multi-omics and high-plex imaging have shown that TAM programs are not randomly distributed but instead align with anatomically and metabolically distinct tumor compartments. Regional sampling bias can shift inferred TAM states^29^. TAMs in tumor cores often display stronger immunosuppressive features, being exposed to extreme metabolic stress—hypoxia, low pH, and nutrient deprivation—that drives HIF-1α stabilization and glycolysis; by contrast, margin-associated TAMs are shaped less by extreme hypoxia/acidosis and more by tissue-injury-associated cues, cytokine networks, and direct interaction with tumor cells undergoing invasion and remodeling. TAMs at invasive margins may retain greater antigen-presenting capacity and interact more with lymphocytes^30^, ^31^. TAMs adjacent to tertiary lymphoid structures (TLSs) can acquire more activated phenotypes that help initiate and sustain antitumor immunity^32^. Recognizing such spatial ecological differences is vital for therapeutic design, since TAMs at different locations can respond very differently to the same strategy.

## 3. Three high-value metabolic axes that shape TAM function

### 3.1 Lactate-pH / hypoxia-HIF-VEGF axis

As a major end-product of glycolysis, lactate accumulates in metabolically active tumors such as BCa and RCC and serves as a key metabolite shaping an immunosuppressive TME^33^-^35^. Lactate affects TAM function and polarization via multiple mechanisms: under hypoxia, intensified glycolysis produces abundant lactate that is exported through MCT1 and MCT4, monocarboxylate transporter proteins. Lactate accumulation and extracellular acidosis stabilize HIF-1α in TAMs, induce ARG1, VEGFA, and PD-L1, and promote polarization toward an M2-like immunosuppressive phenotype^5^. In M1 macrophages, glycolysis is an important metabolic program^36^; downregulation of macrophage glycolysis is consistently accompanied by reduced secretion of inflammatory cytokines^37^. HIF-1α is a well-studied factor that induces glycolysis and M1 polarization, and its overexpression upregulates glycolysis and the pentose phosphate pathway (PPP)^38^.

Lactate is a key immunosuppressive metabolite produced by tumor cells^8^, ^39^, ^40^. Researchers argue that lactate itself, rather than downstream metabolites, drives M2 polarization^41^. In pituitary adenoma, lactate induces M2 polarization via mTORC2 and ERK pathways, after which M2 macrophages secrete CCL17 to promote invasion^42^. Similar mechanisms may exist in urologic cancers, particularly in highly glycolytic BCa subtypes. Upon polarization, macrophage function is affected by lactate: lactate downregulates the macrophage-specific vacuolar ATPase subunit ATP6V0d2, enhances HIF-2α-mediated vascular endothelial growth factor (VEGF) production, and promotes cancer growth^43^. In BCa, TAMs around necrotic cores upregulate MCT4 and CD39, suggesting a lactate-driven adenosine-mediated immunosuppression. Spatial transcriptomics corroborates the co-localization of lactate-enriched regions with immunosuppressive TAM subsets^44^.

Acidic microenvironments driven by lactate can suppress antigen processing and presentation through multiple mechanisms. As reviewed by Wang *et al.*, acidosis undermines DC/macrophage antigen presentation^45^. Furthermore, both *in vitro* and *in vivo*, macrophages are more prone to IL-4-induced M2 polarization at pH 6.8; conversely, neutralizing low pH can shift TAM phenotypes toward inflammatory states and suppress tumor progression^46^. In addition, recent reviews summarize how lactate in the TME promotes protumoral macrophage polarization through metabolic control and signaling pathways such as GPR81^39^, ^47^.

### 3.2 Lipid rafts axis

Lipid metabolic rewiring is a hallmark of urologic cancers—especially PCa and RCC^48^-^50^. Through lipid and cholesterol metabolism, tumor cells and TAMs develop complex metabolic coupling: TAMs dynamically shape immune signal transduction by modulating lipid and cholesterol metabolism^51^, and lipid rafts play a central role in determining immunosuppressive versus activating phenotypes^52^, ^53^.

Lipid rafts, cholesterol- and sphingolipid-rich microdomains, act as signaling platforms that regulate receptor clustering, downstream phosphorylation, and vesicular transport. In TAMs, altered raft composition remodels receptor topology and biases signaling toward immunoregulatory states.

At the transcriptional level, sterol regulatory element-binding protein 1 (SREBP1) acts as a central node linking lipid availability to the immunosuppressive fitness of TAMs^54^, ^55^. In tumors, regulatory T cells can indirectly sustain an immunosuppressive TAM program by maintaining SREBP1-dependent lipid metabolic pathways in macrophages, thereby supporting mitochondrial integrity and survival of protumor TAM states, which provides a concrete rationale for discussing SREBP1 not only as a tumor-cell lipogenesis driver but also as a macrophage-intrinsic regulator of TAM persistence and function in lipid-rich urologic TMEs^55^.

Recent studies indicate that, in hepatocellular carcinoma, cytokine-induced CD36 promotes recruitment of circulating monocytes via CCL2/CCR2/p110γ signaling, thereby expanding TAM populations; at the same time, raft stabilization is reinforced, promoting lipid accrual^56^. These raft-enriched complexes strengthen PI3K-AKT inhibitory signaling and push macrophages toward M2-like polarization^57^. Conversely, LXR-ABCA1/ABCG1-controlled cholesterol efflux governs raft cholesterol content and membrane order^58^. Importantly, tumor settings can exploit this axis in different directions. A study indicates that cancer cells drive cholesterol efflux from TAMs, leading to membrane lipid-raft depletion and a reprogrammed TAM state that favored protumor behavior^59^. This highlights that raft remodeling is pathway-selective—altering cytokine signaling and immune polarization by changing membrane organization rather than simply increasing or decreasing the density of rafts. In parallel, ABCA1-dependent membrane remodeling has been shown to regulate Toll-like receptor signaling by influencing receptor trafficking into rafts, supporting the concept that cholesterol handling can reshape innate immune responsiveness^60^. Such raft-centric reprogramming is particularly pronounced in PCa, where androgen-driven lipogenesis is closely intertwined with macrophage cholesterol metabolism^61^; thus, raft-macrophage metabolic coupling is also likely to manifest across urologic tumors. Spatial metabolomics reveals higher abundances of metabolites related to lipid synthesis and remodeling in PCa compared with normal prostate^62^. Lipid rafts are not static scaffolds but metabolically responsive decision nodes that govern TAM polarization. Targeting raft dynamics therefore emerges as a potential strategy to reprogram macrophage fate in lipid-enriched urologic tumors.

### 3.3 Ferroptosis-redox axis

Ferroptosis, a newly recognized form of cell death, usually features massive iron accumulation and lipid peroxidation; its occurrence is iron-dependent^63^. Ferroptosis inducers reduce intracellular antioxidant capacity and lead to accumulation of lipid reactive oxygen species (ROS), ultimately causing oxidative cell death^64^, ^65^. Recent research links ferroptosis to multiple diseases, including cancer, neurological disorders, ischemia-reperfusion injury, kidney injury, and hematologic conditions^63^.TAMs import iron through TfR1 and DMT1 and export iron via FPN; the balance determines the level of the labile iron pool. Excess Fe2^+^ triggers in a Fenton-type radical chemistry using iron as the catalyst to generate ROS and initiate membrane lipid peroxidation. M2-like TAMs, which have lower GPX4 and GSH, are more sensitive to ferroptotic stress^11^.

GPX4, as a central enzyme that reduces phospholipid hydroperoxides, can buffer ferroptotic stress and mitigate the toxic effects of ferroptosis. Importantly, macrophage susceptibility to GPX4 loss is activation-state dependent: alternatively activated (IL-4-driven) macrophages become highly vulnerable to ferroptosis when GPX4 is impaired, whereas inflammatory macrophages can be comparatively protected, highlighting ontogeny/activation cues as determinants of ferroptosis sensitivity^66^. Consistent with this, macrophage subsets can display heterogeneous ferroptosis susceptibility linked to differential GPX4 stability and redox wiring, suggesting that ferroptosis-based strategies may selectively pressure M2-like TAM programs but require careful control to avoid collateral impairment of myeloid antigen-handling functions^67^.

From the perspective of ferroptosis substrates, ACSL4 shapes ferroptosis sensitivity by channeling polyunsaturated fatty acids into peroxidation-prone phospholipids. In tumors, cytotoxic T cell-derived IFN-γ can synergize with selective fatty acids to promote ACSL4-dependent tumor ferroptosis, providing a mechanistic basis for how immune activation and ferroptotic vulnerability can reinforce each other during immunotherapy^68^. Emerging data further suggest that ACSL4 can couple ferroptosis propensity to macrophage functional states, including contexts where ACSL4-associated lipid remodeling aligns with M1 polarization^69^-^71^.

When immunogenic ferroptosis occurs, oxidized phospholipids and damage-associated molecular patterns (DAMPs) such as HMGB1 are released, activating macrophages and dendritic cells and promoting cross-presentation^72^, potentially driving TAMs toward M1 phenotypes^73^. In PCa, specific circular RNAs can induce ferroptosis in TAMs by suppressing HSP90, thereby promoting M1-like polarization and hindering tumor progression^74^, ^75^.

Iron metabolism is tightly linked to TAM clearance and cross-presentation capacity^76^. Iron-rich M1-like macrophages often show stronger phagocytosis, but excessive iron may drive ROS overproduction and functional impairment^77^. Under iron restriction, M2-like macrophages tend to express high ferritin and iron-export proteins, maintain low intracellular iron, and support tissue repair and remodeling^76^. Thus, tuning iron metabolism may be an effective strategy to reprogram TAM function, particularly in “cold” tumors enriched with M2-like TAMs.

**Figure 1:**
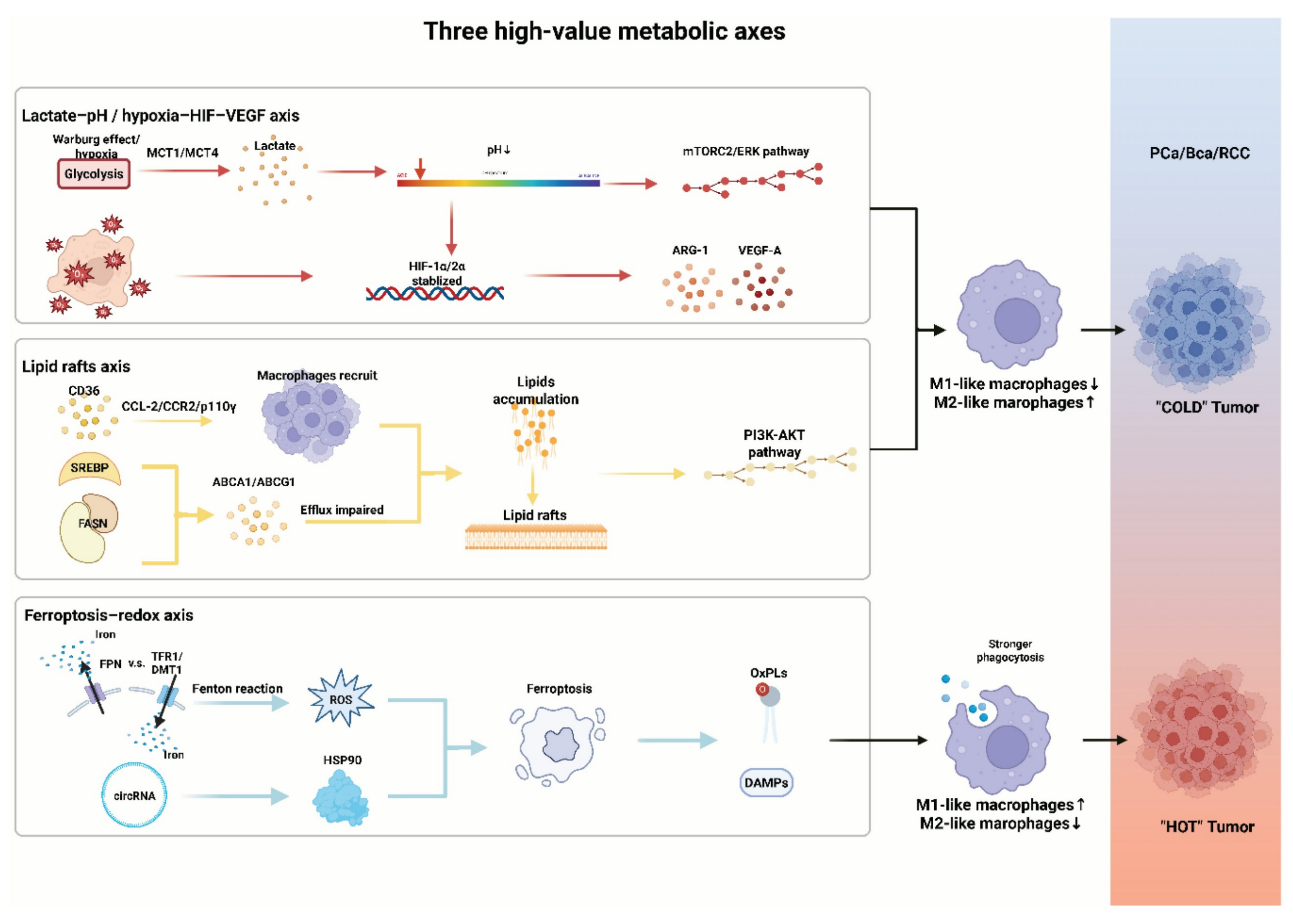
** Three high-value metabolic axes that shape TAM function.** pH reduction and HIF-VEGF axis activation driven by hypoxia and lactate, along with lipid rafts-centered metabolism promotes TAM polarization; ferroptosis triggered by ROS and specific circRNAs drives macrophage polarization and alters the antigen-presenting capacity. MCT, monocarboxylate transporter; HIF, hypoxia-inducible factor; mTORC, mechanistic target of rapamycin complex; ERK, extracellular signal-regulated kinase; ARG-1, arginase-1; VEGF-A, vascular endothelial growth factor -A; SREBP, sterol regulatory element-binding protein; FASN, fatty acid synthase; PI3K, phosphatidylinositol 3-kinase; AKT, ak strain transforming; TFR1, transferrin receptor 1; DMT1, divalent metal transporter 1; FPN, ferroportin; ROS, reactive oxygen species; HSP, heat shock protein; OxPLs, oxidized phospholipids; DAMPs, damage-associated molecular patterns.

This metabolic axis is especially critical in urologic cancers, which frequently involve lipid and iron dysregulation (Figure 1). In the future, combining ferroptosis inducers with ICIs may achieve synergistic clinical translation by acting on both tumor cells and TAMs (Table 1).

Collectively, the lactate-pH/hypoxia-HIF-VEGF axis, lipid rafts signaling, and ferroptosis-redox axis pressure form a convergent mechanistic framework through which metabolic stress is translated into durable TAM programs in urologic tumors. Across these axes, metabolites and membrane organization jointly reshape macrophage polarization, antigen handling, angiogenic output, and immune-checkpoint tone. Key gaps remain in defining how these axes intersect in space and time. Translationally, these insights argue for mechanism-anchored metabolic priming to re-educate TAMs and sensitize tumors to classic treatments, ideally guided by pharmacodynamic and spatial readouts that report on axis engagement and TAM-state transitions.

## 4. Metabolism-epigenetics-immunity triad in TAM plasticity

### 4.1 Metabolites serve as epigenetic regulators

Metabolites serve as substrates or cofactors for epigenetic modifications and directly shape TAM transcriptional programs and functional states.

In breast-cancer models, tumor-secreted arginine is taken up by TAMs and aberrantly activates polyamine biosynthesis. Excess spermidine modulates DNA demethylase activity, remodeling the epigenetic states of key immune-regulatory genes and ultimately driving macrophages toward immunosuppressive phenotypes^78^.

As noted, hypoxia stabilizes HIF-1α, which induces glycolysis and M1 polarization^38^. Beyond the canonical HIF pathway, hypoxia elicits profound epigenetic reprogramming. By altering H3K4me3 distribution, hypoxia drives widespread transcription start site (TSS) switching, thereby reshaping the 5'-UTR of mRNAs. This remodeling changes the translational efficiency of key metabolic genes such as PDK1, facilitating glycolytic reprogramming and enabling cells, including TAMs, to adapt to hypoxia^79^.

Microbiome-derived metabolites also participate in these feedback circuits. For example, indole-3-acetic acid produced by the commensal Parabacteroides distasonis suppresses fatty acid synthase (FASN) expression via the AhR axis, thereby promoting phospholipid remodeling and increasing ferroptosis sensitivity in BCa cells. Such metabolism-epigenetic reprogramming directly reshapes the TME and provides a new perspective for targeting microbe-host interactions^80^.

### 4.2 Metabolically controlled transcription factors

Multiple transcription factors act as metabolic sensors connecting cellular metabolic states to TAM gene programs. HIFs are stabilized under hypoxia but are also regulated by metabolites such as succinate and fumarate, driving genes for angiogenesis and glycolysis. In FH-deficient RCC, fumarate accumulation inhibits HIF prolyl-hydroxylases, stabilizes HIF, and promotes angiogenesis and tumor progression^28^.

PPARs, regulated by lipid metabolites, are highly expressed in M2-like TAMs and promote fatty-acid oxidation and oxidative phosphorylation (OXPHOS)^81^. SREBPs, as master regulators of cholesterol and fatty-acid synthesis, are activated in lipid-loaded TAMs, supporting membrane biogenesis and raft formation^82^, ^83^. NRF2 responds to oxidative stress and regulates antioxidant networks, protecting TAMs against ROS damage^84^. Members of the STAT family are activated by cytokines and growth factors: STAT1 drives M1-associated genes, whereas STAT3/STAT6 foster M2 polarization^81^.

### 4.3 Metabolically guided immune outputs

Metabolic reprogramming and the resultant epigenetic changes ultimately modulate cytokine/chemokine outputs, fixing TAMs into distinct functional states that directly influence antitumor immunity.

Work by Kuang *et al.* in HCC demonstrates that the tumor-exported polyamine metabolite N1-acetylspermidine induces macrophage polarization toward immunosuppressive states and recruits CCR8^+^ regulatory T cells, thereby diminishing the efficacy of checkpoint blockade^85^. This is a prototypical case in which metabolites directly govern immune-cell function and output into specific immune patterns.

A review by Malczewski, A. B *et al.* details how gut microbial metabolites optimize immune responses. Short-chain fatty acids enhance CD8^+^ cytotoxicity and memory differentiation by inhibiting histone deacetylases and activating GPCRs, thus augmenting ICI efficacy. This indicates that metabolic signals from distant organs (the gut) can, via epigenetic mechanisms, systemically influence local tumor immunity^86^.

### 4.4 Metabolism-epigenetics-immunity feedback loops

Within the TME, TAM metabolism-epigenetics-immunity states are not isolated; they are continuously shaped by signals from other cells and systems. Through cytokine and chemokine production, the metabolic state of TAMs forms complex feedback loops with other TME components.

As noted, inflammatory macrophages stimulate hepatocellular carcinoma cells to upregulate spermidine/spermine N1-acetyltransferase 1, inducing production and export of N1-acetylspermidine. This metabolite in turn acts on macrophages, polarizing them toward more immunosuppressive phenotypes—constituting a malignant positive feedback loop mediated by metabolites between tumor cells and TAMs that progressively exacerbates immunosuppression^85^.

For example, IL-10 and TGF-β produced by M2 TAMs not only directly suppress T-cell function but also influence metabolic properties of tumor and other immune cells to promote tumor progression^5^. Likewise, tumor-derived metabolites such as lactate and adenosine further reinforce immunosuppressive TAM phenotypes, forming self-sustaining feedback circuits^25^, ^43^, ^87^.

## 5. Disease-specific syntheses

### 5.1 Prostate cancer / castration-resistant prostate cancer (CRPC)

Prostate tumors—especially CRPC—exhibit high dependence on lipid synthesis and uptake. This metabolic remodeling affects tumor cells and TAMs, while cholesterol-rich lipid rafts organize immunoregulatory and motility signals on membranes^10^, ^88^, ^89^.

Lipid addiction in CRPC commonly features SREBP-driven lipogenesis and upregulation of fatty-acid uptake/desaturation programs such as FASN, SCD, which support proliferation, stress tolerance, and therapeutic resistance^90^. Accumulation of cholesterol and complex lipids not only satisfies biosynthetic needs but also impacts antigen presentation and cytokine secretion in myeloid cells within the microenvironment^88^, ^91^. Cholesterol-enriched rafts act as “signal islands” that co-localize receptors such as AR complex, TRPM8 and kinases to promote tumor-cell survival and motility; raft disruption alters phosphorylation cascades and weakens these signals^10^, ^89^, ^92^. Such raft-organized platforms are also associated with immune evasion because raft composition modulates myeloid-receptor clustering and downstream PI3K-AKT/TREM2-linked inhibitory programs^93^.

In the TME, accumulations of Myeloid-derived suppressor cells (MDSCs) and TAMs become key drivers of androgen receptor (AR)-independent resistance. For example, in microenvironments with high Wnt5a, infiltration of CD68^+^ macrophages increases significantly; these induced macrophages secrete IL-10 and TGF-β, directly suppressing CD8^+^ T-cell function and driving immunosuppression^94^—revealing a resistance pathway mediated by myeloid cells that bypasses AR signaling itself. Macrophages may also promote anti-androgen resistance via paracrine loops and AR/TREM2 signaling within myeloid cells, collectively weakening antitumor immunity and accelerating progression^95^, ^96^.

**Figure 2:**
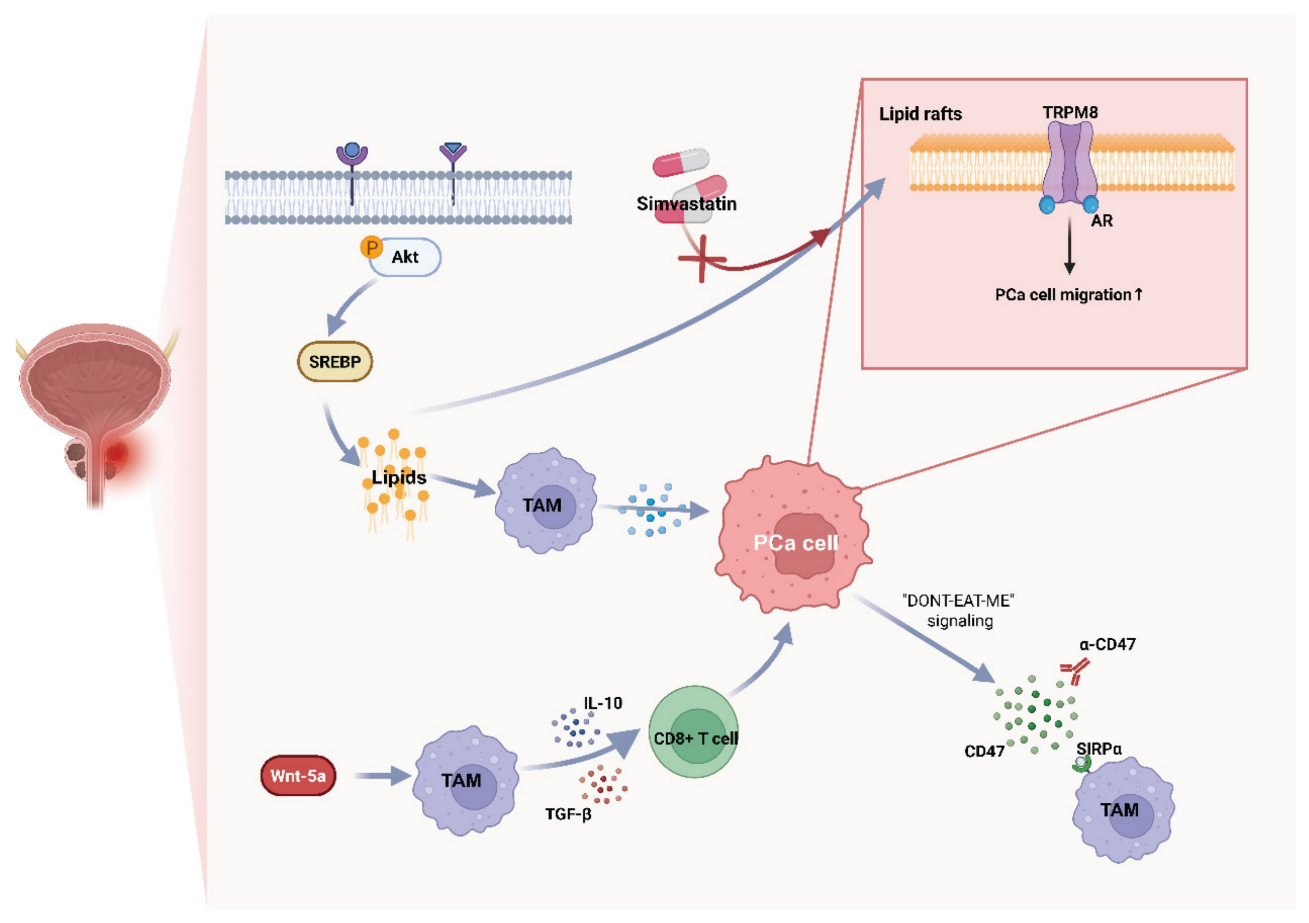
** Representative metabolic-immune coupling pathways in prostate cancer.** Lipid Rafts-stabilized inhibitory signaling and AR-independent myeloid circuits Wnt-5a blunt macrophage functions; in NEPC, upregulation of CD47-SIRPα axis suppresses clearance. TAM, tumor-associated macrophage; TRPM8, transient receptor potential melastatin 8; SIRPα, signal-regulatory protein α.

In addition, neuroendocrine transdifferentiation (NEPC) is a common event in PCa progression and is associated with distinctive metabolic and immune features. Therapeutic pressure can drive lineage plasticity toward neuroendocrine phenotypes through factors such as activation of FOXA2, ONECUT2, and KIT, resulting in AR ^low^/negative states and altered immune interactions^97^, ^98^. NEPC and advanced CRPC often upregulate “don't-eat-me” checkpoints—most notably CD47—which engages macrophage SIRPα to suppress phagocytosis; blocking CD47/SIRPα restores clearance and enhances antitumor immunity in preclinical systems^99^-^101^. Moreover, reverse signaling through CD47 may support PCa-cell survival under phagocytic stress, suggesting a dual role for this axis in lineage plasticity and refractory disease^102^ (Figure 2).

### 5.2 Bladder cancer: glycolytic and adenosinergic immunosuppression

As noted, BCa is characterized by high glycolytic activity and lactate accumulation, creating an acidic, adenosinergic milieu that polarizes TAMs, weakens T/NK-cell effector programs, and maintains an immune-excluded (“cold”) phenotype^103^, ^104^.

In BCa and other solid tumors, lactate accumulation biases myeloid cells toward suppressive states (e.g., Arg1^high^/IL-10^high^) and impairs cytotoxic lymphocytes; new studies suggest lactate consolidates immune paralysis via epigenetic reprogramming such as histone acetylation/lactylation^105^, ^106^. In BCa cohorts, glycolysis signatures correlate with poorer prognosis and immune imbalance, supporting glycolysis as a driver rather than a bystander^106^.

Adenosinergic immunosuppression is another hallmark of the BCa TME. Under hypoxia and cellular stress, CD39 and CD73 are upregulated on tumor/stromal compartments (including BCa-derived exosomes), converting ATP to adenosine; adenosine signals through A2A/A2B receptors to raise cAMP and blunt antitumor responses^107^, ^108^. In the syngeneic MB49 model, pharmacologic A2B blockade slows tumor growth; recent orthotopic bladder cancer studies show that co-targeting CD73 augments anti-PD-L1 efficacy, nominating the adenosine axis as an actionable immunosuppressive hub in BCa^109^, ^110^. Notably, TAMs themselves are an important source of adenosine in urologic tumors, creating self-reinforcing immunosuppression^111^.

Recent data indicate the potential to “warm” cold tumors by targeting these metabolic axes.

**Figure 3:**
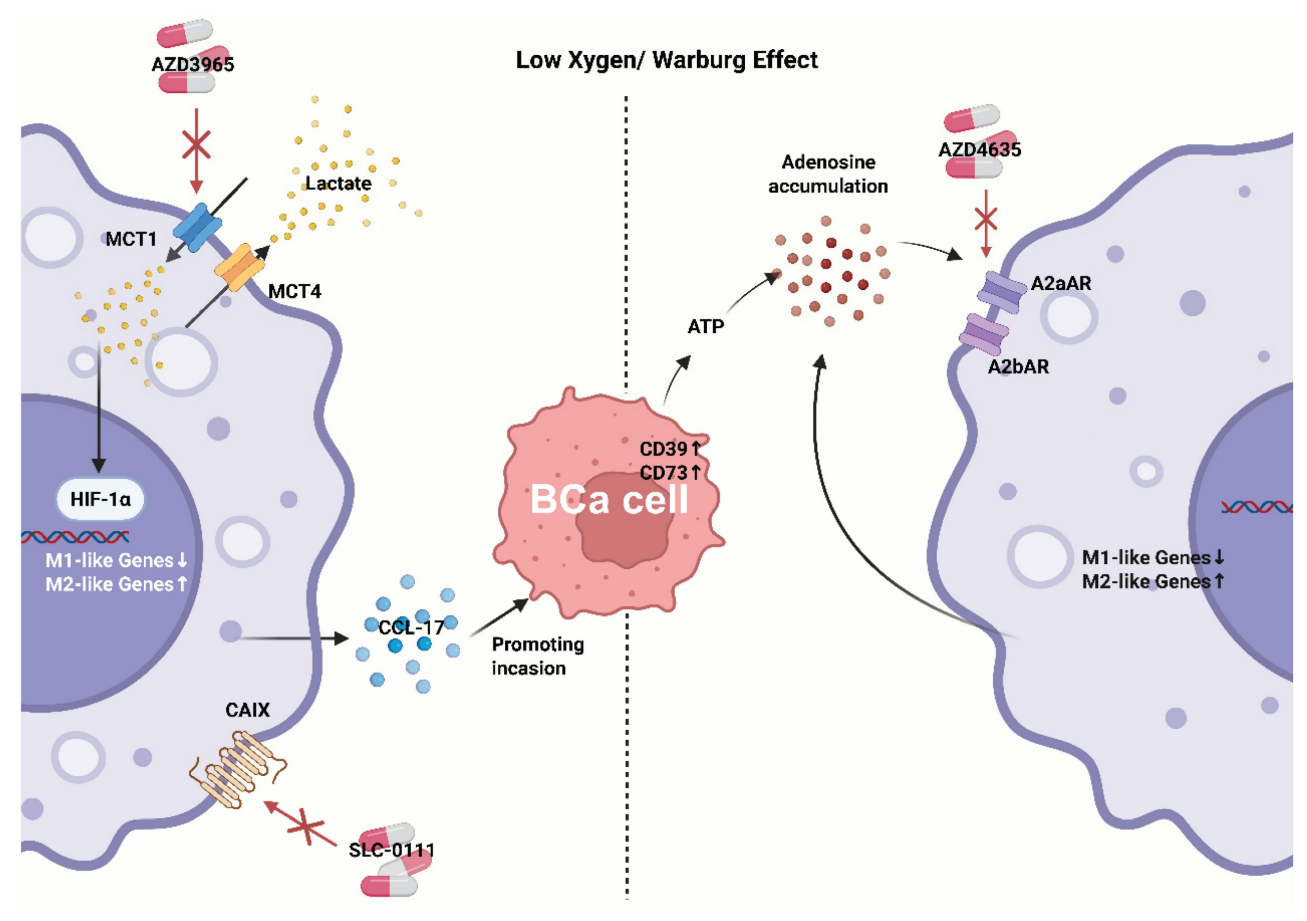
** Representative metabolic-immune coupling pathways in bladder cancer.** Acidic/adenosinergic niches suppress antigen handling and promote TAMs polarization, maintaining a "cold" phenotype despite checkpoint blockade. CAIX, carbonic anhydrase IX.

(1) **Lactate control (production/transport).** Inhibiting LDHA or blocking MCT1/4-mediated lactate shuttling increases metabolic stress in tumor cells while reducing immunosuppressive export^112^. The MCT1 inhibitor AZD3965 has first-in-human data, and preclinical evidence for LDH blockade is accumulating^113^. In BCa models, LDHA activity associates with invasiveness; mechanistically, combining lactate-pathway inhibitors with ICIs may restore antigen presentation and CTL function within acidic niches^104^, ^114^.

(2) **Purinergic blockade (CD39/CD73/A2A/A2B)**. Strategies include anti-CD39 or anti-CD73 antibodies and small-molecule A2A/A2B antagonists; across multiple tumors, such agents release the adenosine “brake” and synergize with PD-1/PD-L1 therapy, with orthotopic BCa evidence as disease-specific support^109^, ^115^, ^116^.

(3) **Integration with intravesical immunotherapy.** BCG relies on strong innate recognition and trained-immunity-like programs; in principle, tuning lactate and adenosine could augment BCG-induced myeloid activation and cross-priming, potentially converting partial responders into inflamed responders^117^-^119^ (Figure 3).

### 5.3 Renal cell carcinoma: HIF-centric metabolism and angiogenesis coupling

RCC—particularly the clear-cell subtype (ccRCC)—is characterized by early loss of **VHL**, with consequent stabilization of **HIF-α**, which drives profound metabolic reprogramming. This includes heightened glycolysis, diminished OXPHOS, and lipid-droplet accumulation, profoundly influencing the TME and producing a lipid-laden phenotype^120^-^123^.

Lipid droplets serve as metabolic hallmarks. ccRCC accumulates neutral lipids and cholesteryl esters in droplets driven by HIF-regulated programs such as HILPDA/PLIN family and glutamine-dependent lipogenesis; droplets buffer redox stress and supply fatty acids for signaling and membrane remodeling^124^, ^125^. Lipid-rich tumor cells also alter myeloid metabolism via lipid mediators/oxylipins and impair antigen presentation; TAMs exposed to HIF-driven lipid signals readily acquire pro-angiogenic, immunoregulatory states^126^-^128^.

TAM-angiogenesis coupling plays a central role in RCC progression. TAMs produce pro-angiogenic factors, including VEGF-A and ANGPT2; via the VEGF/ANG-TIE2 axis, they collaborate with tumor-derived angiogenic signals to remodel endothelium and recruit Tie2^+^ macrophages (TEMs)^129^, ^130^. Although neovascularization supplies oxygen and nutrients, it is typically abnormal and leaky, exacerbating hypoxia and acidosis and creating self-reinforcing loops^131^. Anti-VEGF therapy is standard-of-care in RCC, and part of its efficacy depends on effects on TAMs. Anti-VEGF/VEGFR Tyrosine kinase inhibitors (TKIs) can transiently normalize vasculature, facilitate Cytotoxic T lymphocyte (CTL) infiltration, and modulate myeloid composition—considered a mechanistic basis for ICI-TKI combinations (first-line regimens such as pembrolizumab-axitinib, pembrolizumab-lenvatinib, nivolumab-cabozantinib) that improve OS/PFS^132^-^135^.

**Figure 4:**
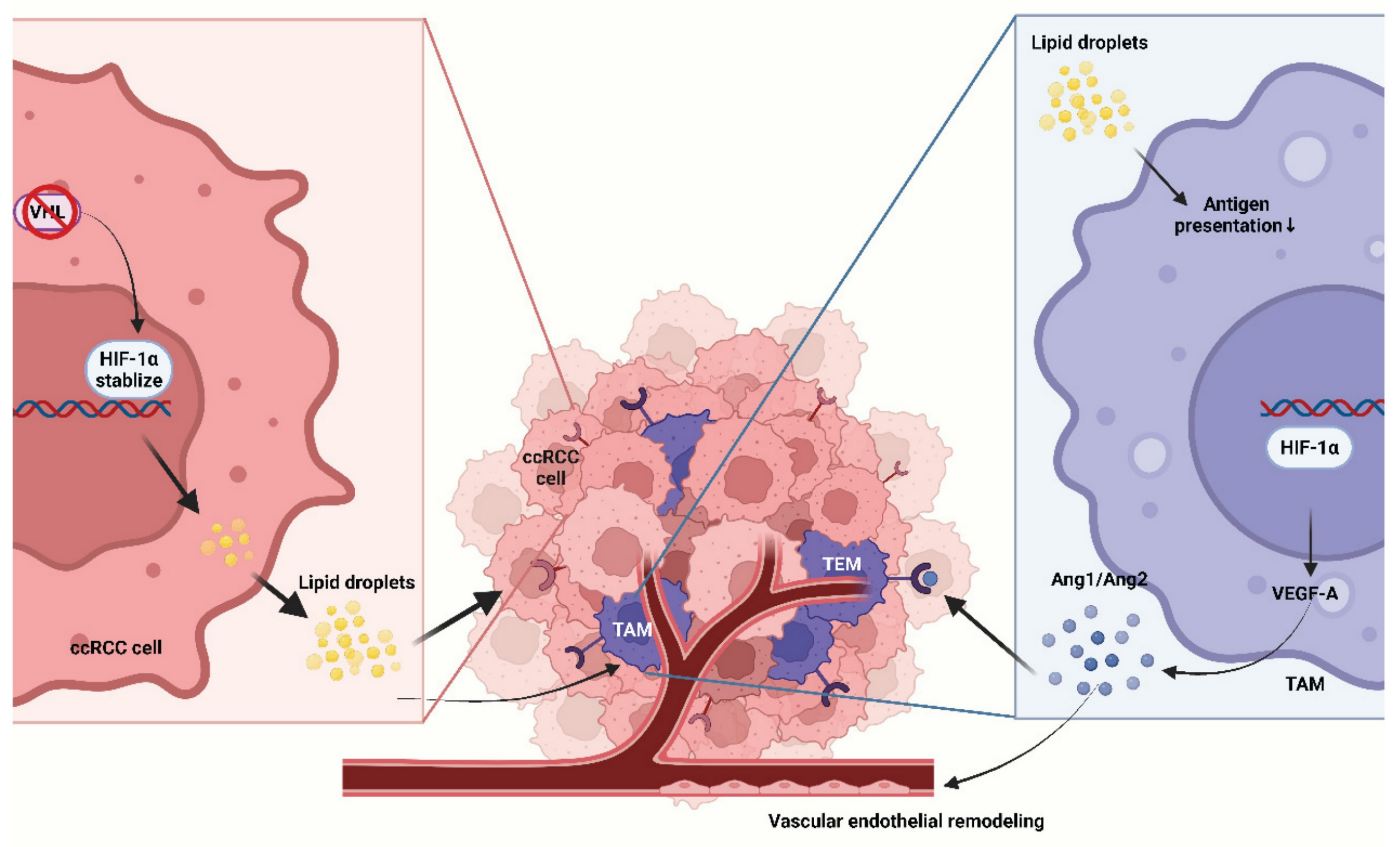
** Representative metabolic-immune coupling pathways in clear cell renal cell carcinomas.** VHL loss leads to HIF signaling activation, manifested as lipid droplet accumulation that shapes a lipid droplet-rich clear-cell phenotype; cytokines produced by TAMs act through the VEGF/ANG-TIE2 axis and synergize with tumor cell-derived angiogenic signals to remodel the endothelium and recruit TEMs. Ang, angiopoietin; TEM, Tie2-expressing macrophage.

Targeting upstream HIF, hypoxia also enhances immune suppression and sustains TAM programs that promote aberrant vasculature, providing a mechanistic rationale to pair HIF-2α blockade with PD-1/PD-L1 inhibitors^136^-^138^. Cutting-edge research is now further exploring the feasibility of combining HIF-2α inhibitors with ICIs. Clinically, the phase 3 LITESPARK-005 trial established belzutifan (HIF-2α inhibitor) activity after prior PD-(L)1 and VEGF-TKI: PFS HR 0.75 (95% CI 0.63-0.90) versus everolimus, a mTOR inhibitor; due to non-proportional hazards, medians were similar (both 5.6 months), yet curves separated late and responses were higher for belzutifan^139^. On-target toxicities of HIF-2α inhibition such as anemia, hypoxia are predictable and manageable with dose modification and monitoring, an important consideration when layering with ICI. Belzutifan and Lenvatinib combination has shown promising activity post-PD-1/PD-L1 in multicenter studies and press-release analyses, meeting PFS and ORR endpoints versus cabozantinib in previously treated disease, showing combination strategies are maturing^140^ (Figure 4).

Disease-specific metabolism selects for distinct macrophage programs and resistance logics that can be mapped onto the three metabolic axes rather than simply reprogram TAMs in urologic cancers. Harmonizing cross-cancer TAM taxonomies remains gaps needed to be further studied. While another gaps is the limited ability to prospectively identify which metabolic niche is rate-limiting in an individual patient, especially given strong spatial heterogeneity. Translationally, these points argue for organ- and niche-tailored strategies rather than uniform TAM targeting, with patient selection and response evaluation anchored to spatially informed biomarkers.

## 6. Translational Toolkit: Models, Readouts, and Biomarkers

### 6.1 Preclinical model systems

Studying metabolism-immunity crosstalk in urologic tumors requires models that capture complex metabolic coupling between tumor and immune cells.

Organoids with immune co-cultures—co-culturing tumor organoids with autologous or syngeneic immune cells—preserve tumor genomic and spatial heterogeneity along with patient-specific metabolic and immune features. They are suitable for high-throughput screening and mechanistic work and allow controlled manipulation of lactate, cholesterol, and adenosine pathways. Recent reviews and urologic exemplars describe immune-organoid systems that incorporate myeloid components, recapitulate *in-vivo* settings, permit precise control of microenvironmental variables (pH, O2, metabolites), and integrate smoothly with spatial/multiplex readouts to direct TAM perturbation. Organoids have been established across PCa, BCa, and RCC^141^-^143^. Nonetheless, these systems simplify the immune milieu, lack full stromal/vascular signals, and cannot fully encompass the entire metabolism-immunity nexus; long-term culture may induce metabolic drift, limiting predictive value^143^.

Humanized xenograft models implant human tumors into immunodeficient mice reconstituted with human immune systems, enabling *in-vivo* assessment of human-specific targets. They are crucial for testing strategies that target human metabolic enzymes or checkpoints, and they provide intact vasculature for combinations such as ICI, anti-CD73/A2A, and HIF-2α inhibitors^144^, ^145^. However, interspecies metabolic gaps, risk of GVHD, and incomplete myeloid reconstitution may impact TAM-focused studies^144^.

*Ex vivo* precision-cut tumor slices preserve native TME architecture and composition and suit short-term readouts for metabolic interventions. Fresh slices from RCC/PCa/BCa retain multicellular structure, gradients, and local metabolite pools, enabling rapid Pharmacokinetics/ Pharmacodynamics (PK/PD) assessment of therapy-induced metabolic and immune functional changes close to the patient context; nonetheless, tissue availability and throughput constrain their use^146^.

Each model has unique strengths and limitations. Selection should be tailored to the scientific question and hypothesis under test.

### 6.2 Readouts: multi-omics and imaging

Advanced analytics provide unprecedented insight into TAM metabolic states and functions.

Metabolic flux analysis uses stable-isotope tracers to track fluxes and reveal authentic in-situ TAM metabolism. When integrated with scRNA-seq and spatial transcriptomics, these data resolve metabolic heterogeneity among TAM subsets and across space^147^-^149^.

Single-cell spatial multi-omics, including spatial transcriptomics and mass spectrometry imaging, offers spatial context for metabolite distributions, cell composition, and gene expression within native tissues. In urologic tumors, integrating these technologies with metabolism-immune phenotypes reveals how metabolic gradients shape TAM distribution/states (phagocytic, angiogenic, regulatory) and their proximity to metabolic niches (acidic, lipid-droplet-rich, hypoxic)^150^-^152^.

Metabolic imaging, such as hyperpolarized MRI and specialized PET tracers, supports noninvasive, longitudinal monitoring of metabolic interventions. For example, HP ^13^C-pyruvate MRI quantifies real-time pyruvate to lactate conversion rate (k_PL_) and is advancing clinically in PCa; molecular PET beyond FDG such as hypoxia and glutamine tracers is applicable to urologic tumors^153^-^156^.

Functional assays such as phagocytosis/ADCP rely on flow cytometry or imaging like pH-sensitive cargo and live uptake to quantify macrophage effector functions linked to antitumor immunity^157^.

### 6.3 Biomarker development

Successful clinical integration of metabolism-immune targeting requires biomarkers that identify likely responders. Representative candidate biomarkers include ANGPT2, HP ^13^C-pyruvate MRI k_PL_, myeloid-derived IL-23, and PLIN2.

**6.3.1 Plasma ANGPT2 (RCC)** is a myeloid-angiogenesis coupling biomarker associated with ICI-TKI efficacy. ANGPT2 rises during anti-VEGF escape and recruits/perivascularly localizes Tie2^+^ macrophages, strengthening sprouting angiogenesis and immunosuppression^158^, ^159^. High ANGPT2 often corresponds to abnormal, leaky vasculature; on-treatment ANGPT2 decline parallels vascular normalization, facilitating CTL entry (frequently observed under VEGFR-TKI + ICI)^160^. The ANGPT2/TIE2 module resonates with TEM (Tie2^+^ macrophage) transcriptional features and the CSF1/IL-34-CSF1R axis, highlighting an immune-vascular network^160^. Thus, plasma ANGPT2 should be correlated with TEM density and endothelial tip-cell features in tumor slices/spatial omics and can serve as a PD readout to monitor early responses to ICI—TKI combinations. Because nonspecific inflammation can also elevate ANGPT2, it must be interpreted alongside perfusion/hypoxia imaging indices and myeloid gene modules.

**6.3.2 HP ^13^C-pyruvate MRI k_PL_ (PCa & BCa):** Hyperpolarized (HP) ^13^C-pyruvate MRI is a stable-isotope molecular imaging modality that provides real-time assessment of the rate of metabolism through glycolytic pathways in human prostate cancer. HP ^13^C-pyruvate MRI k_PL_, as a live glycolytic-flux biomarker associated with “cold” to “hot” transitions, reflecting glycolytic pressure that shapes acidity/adenosine niches and TAM polarization^161^. In metastatic PCa, k_PL_ can decline early on therapy and correlate with metabolic response; in metabolism-immunity studies, a drop in k_PL_ can serve as an indirect readout of lactate-pathway inhibition (LDH/MCT) or ICI engagement during transition from “cold” to “hot” phenotype^161^, ^162^. It is suitable as a PD biomarker and early surrogate endpoint, for example, k_PL_ decline linked to changes in urinary/blood lactate and adenosine metabolites. Although evidence for individual stratification is still accruing. Meanwhile, sensitivity is limited for lesions not dominated by glycolytic metabolism^161^, ^163^.

**6.3.3 Myeloid-derived IL-23 (CRPC):** a driver of AR-independent resistance and a serum/tissue biomarker. IL-23 secreted by MDSCs sustains CRPC growth and AR program output under androgen-deprived conditions; blockade reverses this effect in animal models and human explants. Elevated IL-23 often coexists with myeloid infiltration and exhausted T cells; IHC/multiplex imaging shows pSTAT3 activation co-localizing with IL-23 in CD68^+^ / CD163^+^ myeloid cells. The IL-23 axis can link NF-κB/STAT3 inflammatory programs with residual AR-target gene expression, indicating a “myeloid—AR-independent resistance” route^164^. Therefore, IL-23 was considered to have potential for predicting anti-androgen resistance and monitoring responses to myeloid-targeted interventions, although prospective thresholds, standardized assays, and interventional trials are needed. Because IL-23 also rises in systemic inflammation, single-analyte specificity is limited; it is best interpreted alongside MDSC/TAM transcriptional signatures and circulating cytokine panels^164^.

**6.3.4 PLIN2 (RCC):** a marker linking HIF-driven lipid-droplet biology to noninvasive urinary detection. PLIN2 coats lipid droplets and is upregulated in VHL/HIF-driven ccRCC; urinary PLIN2 shows good diagnostic performance. Histologically, PLIN2 immunocytochemistry (IHC) aligns with the “clear-cell/lipid-droplet-rich” phenotype; metabolic imaging often shows expanded lipid pools and rich perfusion (relevant for anti-angiogenic treatment selection and monitoring)^165^. PLIN2 co-upregulates with HIF targets and lipid-droplet programs, delineating a HIF-centric lipid-droplet axis^166^. It is suitable for noninvasive screening/differential diagnosis and follow-up monitoring; however, dedicated studies on predictive value for immune combinations are lacking, and combined interpretation with imaging/gene programs is most prudent^165^.

#### 6.3.5 Summary

In general, circulating biomarkers offer accessible alternatives but may not fully mirror site-specific metabolism-immunity interactions; imaging biomarkers provide whole-tumor assessment and noninvasive monitoring but may lack molecular specificity. An ideal strategy likely integrates multi-layered information to maximize predictive value while minimizing invasiveness and cost (Table 2).

## 7. Therapeutic strategies and combination

In urologic cancers, metabolic reprogramming shapes immunosuppressive ecologies—acidic, adenosinergic, and raft-stabilized—that push macrophages toward tumor-promoting states; accordingly, clinically relevant treatments increasingly turn to direct metabolic interventions and immunotherapy combinations to re-educate macrophages and restore cytotoxic programs.

### 7.1 Direct metabolic interventions

Because lactate and low pH blunt antigen presentation and suppress macrophage phagocytosis, lactate-pathway interference is attractive. The clinical MCT1 inhibitor AZD3965 demonstrated acceptable safety and on-target pharmacodynamics in first-in-human studies, raising intracellular lactate and remodeling tumor bioenergetics as expected; these data provide a feasible foundation for lactate relief + ICI treatment^113^. In parallel, tumor acidity buffering with systemic alkalinizing agents such as bicarbonate/THAM) has long been shown in preclinical models to increase intratumoral pH and improve drug penetration, yet widespread clinical adoption requires rigorous standardization of dose, patient selection, and safety frameworks^167^-^169^. Carbonic anhydrase IX (CAIX) is a transmembrane protein closely associated with the hypoxic conditions of the tumor microenvironment. Given CAIX's strong hypoxia-link and overexpression in RCC and a subset of bladder cancers, CAIX blockade remains a rational direct approach to neutralize extracellular acidity that impairs drug penetration and myeloid function^170^. The CAIX inhibitor SLC-0111 completed a phase-I trial with acceptable tolerability and pharmacokinetics across solid tumors, operationalizing pH-axis targeting in humans^171^. As previously mentioned, hypoxia and lactic acid accumulation contribute to the reinforcement of the M2-like TAM phenotype.

These treatments have the potential to ameliorate hypoxia, increase intratumoral pH and has been shown in preclinical models to improve responses to checkpoint blockade; mechanistically, such buffering is expected to relieve pH-driven suppression of macrophage phagocytosis and antigen presentation, thereby facilitating T-cell reinvigoration and potentially supporting “cold” to “hot” transitions.

Lipid and cholesterol modulation offers another promising direction because these programs stabilize suppressive signaling platforms in myeloid and tumor cells. The selective FASN inhibitor denifanstat TVB-2640 has advanced through phase I/II settings with manageable toxicity and combinability, indicating that lipid-anabolism modulation is clinically tractable and may, in principle, reduce de novo lipogenesis that supports suppressive TAM programs in lipid-rich niches; mechanistically, constraining lipid flux and perturbing cholesterol-rich rafts may dismantle myeloid suppressive circuits and enhance ICI sensitivity.^172^ Classic mechanistic work in PCa shows that cholesterol targeting disrupts raft-dependent survival and motility signals—a principle applicable to macrophage-tumor crosstalk where raft clustering coordinates PI3K/AKT and immunoregulatory receptors^10^.

Iron metabolism and ferroptosis modulation can be pursued with iron chelators (e.g., deferoxamine) and ferroptosis inducers (e.g., erastin derivatives). Although iron chelators are pharmacologically mature and may suppress unchecked peroxidation loops in select settings, oncology-specific schedules and on-target immune consequences require prospective validation^173^. Conversely, inducing ferroptosis, by inhibiting system xc- or GPX4, or via nano-delivery, can increase immunogenic stress and antigenicity, but effects on myeloid states are context-dependent and call for careful dose-finding and cautious integration with ICIs^63^, ^174^-^176^. This therapeutic strategy attenuates iron-catalyzed Fenton-type radical reactions, thereby reducing ROS generation and mitigating membrane lipid peroxidation. As previously discussed, while iron-rich M1-like TAMs typically exhibit enhanced phagocytic capacity, excessive iron levels can trigger ROS overproduction, leading to functional impairment. Conversely, under iron-restricted conditions, M2-like TAMs prioritize the expression of ferritin and iron exporters to maintain low intracellular iron levels, which supports their role in tissue repair and remodeling. Consequently, modulating iron metabolism and ferroptosis represents a promising strategy for reprogramming TAM functions, particularly within 'cold' tumors characterized by an abundance of M2-like TAMs.

### 7.2 Combination strategies

Combining metabolic interventions with ICIs shows great promise in preclinical models. The rationale is that metabolic reprogramming can convert “cold” tumors into “hot” tumors and thus enhance ICI efficacy.

HIF-2α inhibitor-Belzutifan-based combinations are leading the metabolic-ICI interface in RCC, with multiple readouts now available across lines of therapy^177^. The phase II LITESPARK-003 (NCT03634540) doublet belzutifan + cabozantinib demonstrated durable antitumor activity in both treatment-naïve and post-immunotherapy cohorts, offering a modular backbone for future ICI-layering or sequencing^177^. In another umbrella KEYMAKER-U03 Substudy 03A (NCT04626479), first-line belzutifan + pembrolizumab + lenvatinib achieved high activity; notably, Arm 4 showed ORR 77.5% (95% CI 66.8-86.1) and median PFS 31.8 months (95% CI 26.3-NR) vs the pembrolizumab + lenvatinib reference (HR 0.45, 95% CI 0.25-0.83), with anemia emerging as the expected on-target toxicity of HIF-2α blockade^178^.

Adenosine pathway and ICIs combinations seek to release hypoxia-linked immunosuppression upstream of PD-1/PD-L1^179^, ^180^. In mCRPC, the phase II modular study AZD4635 (A2A antagonist) + durvalumab and/or oleclumab (D8731C00001/NCT04089553) established feasibility and safety with mixed efficacy, informing patient-selection strategies and biomarker development for adenosine-rich niches^,181^. In MIBC, the neoadjuvant durvalumab ± oleclumab (anti-CD73) feasibility study BLASST-2 (NCT03773666) demonstrated practicality of perioperative adenosine-axis modulation; institutional and registry pages detail design and endpoints while mature efficacy readouts are awaited. In RCC, ciforadenant (CPI-444, A2A antagonist) + atezolizumab (NCT02655822) produced signals of activity in early-phase studies, with exploratory adenosine-gene signatures associating with response—supporting a test-and-treat paradigm for adenosinergic niches^182^. The combination serves as a strategic intervention to abrogate adenosine-mediated myeloid immunosuppression, thereby shifting TAM polarization from IL-10-producing, repair-associated programs toward MHC-II^high^ inflammatory antigen-presenting phenotypes. This phenotypic reprogramming may act synergistically with PD-1/PD-L1 inhibition to facilitate the 'cold-to-hot' transformation of the tumor microenvironment in adenosine-rich tumors. Nevertheless, the clinical translation of this synergy necessitates further validation via prospective TAM characterization within specific urologic malignancy cohorts.

### 7.3 Considerations for clinical development

Safe translation requires giving equal weight to timing, sequencing, and dose-finding. A pragmatic approach is a stage-gated design: begin with a short metabolic run-in like MCT1 or CD73/A2A modulation to document PD effects and tolerability; proceed to add ICIs once predefined PD thresholds are met; and adaptively enrich patients by biomarkers-glycolysis ^high^ (for MCT1 ± ICI), adenosine ^high^ (for CD73/A2A ± ICI), or ANGPT2 ^high^ (for ICI with anti-angiogenic support). Dose escalation should prioritize on-target immune modulation rather than global myelosuppression, with predefined stop rules based on PD readouts (HP ^13^C-pyruvate MRI k_PL_, adenosine/lactate panels, ANGPT2) and safety signals (acid-base balance for buffering, lipid/cardiac panels for FASN inhibition, cytopenias for iron-modifying agents).

At the exploratory-decision stage, correlating soluble metabolites with spatial macrophage phenotypes in organoids/*ex-vivo* slices suffices; at early go/no-go nodes, test-retest HP ^13^C-pyruvate MRI and predefined adenosine panels can anchor decisions; and for registrational use, analytically validated thresholds and multi-analyte composites are likely required.

Broadly, successful clinical translation of metabolism-immunity combinations requires careful attention to timing and sequencing. Preclinical data suggest that in some contexts, metabolic priming should precede immunotherapy to appropriately reprogram the TME, whereas in others, concurrent dosing may yield optimal synergy. Dose-finding is also crucial, since metabolic drugs may have therapeutic windows distinct from those of ICIs; global myelosuppression may constrain feasible regimens.

Despite the rationale for combining metabolic interventions with immunotherapy, resistance is likely to emerge through several non-mutually exclusive routes. First, metabolic redundancy and plasticity can bypass single-node blockade—for instance, high or induced MCT4 expression has been associated with reduced sensitivity to the MCT1 inhibitor AZD3965, consistent with compensatory lactate export capacity^183^. Second, tumors may reactivate parallel immunosuppressive metabolite circuits, such as reinforcing the CD39-CD73-adenosine axis or shifting signaling toward alternative adenosine receptors, thereby sustaining myeloid/T-cell suppression even when one component is inhibited^184^-^186^. Third, interventions intended to leverage ferroptosis/redox stress can select for strengthened antioxidant defense programs, including FSP1-CoQ and GCH1-BH4-linked protection, or mitochondrial redox buffering via DHODH, potentially limiting durable immune reprogramming. Collectively, these considerations argue for incorporating on-treatment pharmacodynamic readouts, including TAM states, to distinguish target bypass from insufficient immune engagement and to guide rational next-line combinations rather than assuming uniform synergy^187^-^190^.

As for the considerations of unique toxicities and safety considerations of metabolic interventions. A key translational caveat of metabolic-immune interventions is that many targets governing TAM states are also indispensable for normal tissue homeostasis. For example, pharmacologic HIF-2α inhibition such as belzutifan frequently induces anemia and can cause clinically significant hypoxia^191^. In parallel, blockade of lactate transport is mechanistically attractive for disrupting lactate-driven immunosuppression, yet first-in-human evaluation of the MCT1 inhibitor AZD3965 revealed dose-limiting toxicities that were largely on-target, including electroretinogram changes/retinopathy and a cardiac troponin rise, supporting the need for baseline eye/cardiac screening and longitudinal monitoring when targeting lactate shuttling in patients^113^. FASN inhibitor TVB-2640 showed a manageable safety profile dominated by reversible skin and ocular adverse events^192^, while adenosine-axis blockade is generally considered tolerable, early clinical signals commonly include fatigue, pruritus, and appetite decrease^180^.

This chapter translates the mechanistic framework into therapeutic options, but it also highlights why clinical benefit cannot be assumed from pathway plausibility alone. Across direct interventions and rational combinations, the shared goal is to relax metabolite-imposed constraints and shift TAMs away from suppressive or angiogenic programs toward states more compatible with antigen presentation and effector T-cell activity; nevertheless, the actual TAM subset engaged by each intervention is likely niche-restricted and remains under-validated in patient samples. Two practical limitations stand out: resistance driven by metabolic redundancy, and toxicities because many metabolic nodes are essential for normal tissues. These points motivate a trial-development mindset in which TAM-resolved pharmacodynamic endpoints, ideally with spatial context, careful sequencing with ICIs, and safety-aware dosing are treated as core design elements, enabling credible testing of whether metabolic interventions can reproducibly promote “cold” to “hot” transitions in urologic cancers.

## 8. Clinical development landscape and future directions

### 8.1 Current trial landscape

Clinically, three partly overlapping corridors have emerged for bringing metabolic control into myeloid-dominated urologic cancers, and each corridor answers a different question rather than repeating the same signal seen in combination studies.

The HIF-2α experience in ccRCC shows a single metabolic node can be drugged and sequenced around ICI/TKI without losing activity. The phase III LITESPARK-005 trial—belzutifan versus everolimus in patients who had already received PD-1/PD-L1 and VEGF-TKI—showed a PFS benefit (HR ≈ 0.75) and higher ORR for belzutifan, with anemia/hypoxia as an expected on-target pattern. The study not only tried to prove synergy, it also proved that metabolic control after immunotherapy is clinically workable in RCC^193^-^195^. By attenuating HIF-2α-dependent hypoxia programs, belzutifan may also dampen hypoxia-conditioned pro-angiogenic TAM/TEM-like states enriched in perivascular/hypoxic niches, which could reduce immune exclusion and facilitate CD8⁺ T-cell infiltration—i.e., a plausible “cold” to “hot” shift, particularly in combination settings^139^, ^196^.

Studies that focus on the adenosine pathway—AZD4635 + durvalumab/oleclumab in mCRPC (NCT04089553) and ciforadenant (CPI-444) + atezolizumab in RCC/mCRPC (NCT02655822)—were designed to see whether lifting an immunometabolic brake could make an ICI-experienced microenvironment signal again. The results showed that in unselected populations, the activity was modest, but exploratory adenosine gene signatures and mandatory on-study biopsies from the AZD4635 trial pinpointed who might benefit. This corridor therefore legitimizes a filter strategy for adenosine- or lactate-heavy niches, instead of universal combination^181^. A2A receptor antagonist is expected to relieve adenosine-driven immunosuppressive signaling in macrophages, thereby potentially shifting adenosine-conditioned suppressive TAM programs toward more antigen-presenting/inflammatory states^9^, ^180^.

First-in-human inhibition of CAIX with SLC-0111 proved that correcting extracellular acidity is pharmacologically achievable and tolerable in solid tumors, which matters for hypoxic RCC/BCa where acidity directly impairs phagocytosis and drug penetration.

In parallel, glutaminase programs—telaglenastat (CB-839) + nivolumab for RCC/melanoma/NSCLC (NCT02771626) and RCC-focused follow-ups—showed disease control and an acceptable safety profile, but only moderate antitumor activity, implying that future urologic cancers trials should either co-select for glutamine-addicted tumors or use glutaminase agents explicitly as microenvironment primers before ICI^197^-^199^. Cabozantinib has been reported to reduce TAM abundance/M2 polarization and promote a more inflamed microenvironment—together hypothesized to lessen myeloid suppression and facilitate CD8⁺ recruitment that still requires TAM-resolved clinical pharmacodynamic validation^200^.

### 8.2 Failure analyses and paths to success

In recent years, some clinical trials targeting metabolism-immunity axes in urologic tumors have not met their primary endpoints. Potential causes include:

(1) Selection lagging behind mechanism. Glycolysis^ high^ or adenosine ^high^ tumors are rarely pre-specified, diluting benefit signals; pragmatic biomarker gates should be prospectively embedded to enrich patients most likely to undergo TAM reprogramming. (2) Endpoints not aligned with immunometabolic pharmacology. In ICI combinations, RECIST remains necessary but insufficient; pairing iRECIST or immune-adapted endpoints with PD matrices (adenosine/lactate panels and soluble ANGPT2) is more likely to capture macrophage-mediated “cold” to “hot” transitions before size changes occur. (3) PK/PD mismatches. Short-half-life purinergic agents or subtherapeutic alkalinization can cause under-exposure precisely during windows when antigen release and myeloid re-education should be maximal; stage-gated run-ins requiring PD proof such as k_PL_ decline before randomization mitigate this failure mode. (4) Limited tissue access. On-treatment biopsies are sporadic, extending the learning cycle; embedding *ex vivo* slices and spatial profiling into early cohorts can directly visualize TAM phagocytic tone, raft disruption, and hypoxia-adenosine relief, thereby bridging PK/PD with outcomes.

A consensus, executable roadmap should establish a short-cycle, iterative loop: start with perturbation-ready models and end with fit-for-purpose decision tools. Practically, patient-derived organoids and *ex vivo* precision-cut slices provide controlled environments for titrating lactate, adenosine, or lipid interventions while directly imaging phagocytosis, antigen presentation, and raft organization. Readouts should include stable-isotope flux, single-cell/spatial multi-omics of myeloid states, and HP ^13^C-pyruvate MRI kPL as a noninvasive PD anchor that links glycolytic pressure to macrophage re-education.

Maturation of fit-for-purpose biomarkers should follow staged development: exploratory associations (organoid/slice—soluble lactate/adenosine and spatial TAM phenotypes) —analytical validation and test—retest (HP ^13^C-pyruvate MRI k_PL_, adenosine/lactate panels)—prospective clinical utility, with macrophage-centric PD thresholds gating escalation of combination therapy.

## 9. Conclusion

Metabolic-immune coupling is emerging as a central determinant of therapeutic outcome in urologic cancers. In this review, we synthesize a TAM-centered framework across PCa, BCa, and ccRCC by highlighting three convergent metabolic circuits—the lactate-pH/hypoxia-HIF-VEGF axis, the lipid rafts axis, and the ferroptosis-redox axis—that can impose durable immunosuppressive or pro-angiogenic macrophage programs and thereby limit responsiveness to immune checkpoint blockade and anti-angiogenic therapy. This perspective emphasizes TAMs as a tractable therapeutic node at which metabolite-imposed constraints may be relaxed to restore phagocytosis, antigen handling, and immune-permissive vascular states. Moving forward, clinical progress will possibly depend less on developing a single dominant metabolic agent and more on institutionalizing metabolism-informed, TAM-anchored trial designs: metabolic or spatial enrichment at entry such as glycolysis/high adenosine/hypoxia modules, early pharmacodynamic confirmation in organoid-immune co-cultures, humanized or *ex vivo* platforms, and stepwise escalation with PD-1/PD-L1 or VEGF-TKI combinations. Fit-for-purpose biomarkers, including hyperpolarized ^13^C-pyruvate MRI (k_PL_), plasma ANGPT2, spatial NT5E/ADORA2A, lipid-droplet markers such as PLIN2, should be integrated prospectively to shorten decision cycles and render negative arms informative. Establishing multicenter standards for tissue handling and spatial profiling, together with transparent sharing of metabolic-myeloid datasets across PCa, BCa and RCC, will be essential to translate macrophage reprogramming from a mechanistic concept into a predictable and durable clinical strategy.

## Figures and Tables

**Table 1:** Metabolic axes that reprogram tumor-associated macrophages in urologic cancers.

Axis	Key nodes / pathways	Targets (examples)	Expected TAM shift	Niche change	Biomarkers (PD/response)	Example agents / modalities	Future directions	Relative clinical trials	Ref.
Lactate-pH / hypoxia-HIF-VEGF	Warburg glycolysis → lactate export (MCT1/4); extracellular acidosis; hypoxia→HIF-1α/2α→VEGF;adenosine crosstalk (CD39/CD73→A2A/A2B)	LDHA; MCT1/MCT4; HIF-2α; CD39/CD73; A2A/A2B	M2-like→M1-like re-education; ↑phagocytosis, ↑antigen presentation; ↓ARG1/IL-10	↓acidity (↑pH); ↓adenosine tone; transient vessel normalization; ↓hypoxia	HP-¹³C-pyruvate MRI kPL; plasma/urine lactate & adenosine panels; soluble ANGPT2 (RCC); spatial NT5E/ADORA2A modules	MCT1 inhibitor AZD3965; systemic buffering (bicarbonate/THAM); anti-CD73 (oleclumab); A2A antagonist (ciforadenant); HIF-2α inhibitor (belzutifan)	Define priming window before/with ICI; adaptive enrichment for glycolysis/adenosine-high tumors; integrate spatial PD with HP-¹³C imaging	NCT01791595NCT02531919NCT03773666NCT02655822NCT05239728	38414647146121
Lipid rafts	Cholesterol/sphingolipid-rich microdomains;ABCA1/ABCG1 efflux;SREBP-driven lipogenesis; TREM2/PI3K-AKT inhibitory hubs	FASN; SREBP; TREM2; ABCA1/ABCG1(efflux/raft fluidity)	M2-immunoregulatory→pro-inflammatory tilt; ↓raft clustering; ↑APC function	↓raft stability on tumor/myeloid membranes; ↓tolerogenic signaling platforms	Lipid-raft transcriptomic modules; lipidomic signatures; spatial PLIN/HILPDA context (RCC)	FASN inhibitor (denifanstat/TVB-2640); cholesterol targeting/efflux enhancers; statin/oxysterol-axis exploration	Drugging raft biology in TAMs; combine with PD-1/PD-L1 after raft destabilization; define fluidity PD	NCT05743621NCT02922764NCT04691375	585975165
Ferroptosis-redox	Iron import (TfR1/DMT1) & export (FPN); GPX4/GSH antioxidant axis; lipid peroxidation; immunogenic ferroptosis (HMGB1/OxPLs)	System xc⁻ (SLC7A11); GPX4; iron chelation;	Context-dependent: induce immunogenic death → ↑cross-presentation/M1 shift;restrain iron overload to avoid dysfunction	↑oxidized lipids in TME (if induced); potential ↑antigenicity; redox normalization with chelation	Oxidized phospholipids; circulating iron indices; redox gene programs; phagocytosis/ADCP function readouts	Erastin/RSL3 derivatives; GPX4 inhibitors; iron chelators (deferoxamine/deferasirox); nano-ferroptosis platforms	Dose-finding to avoid global myelosuppression; pair with ICI in biomarker-defined settings; map myeloid-specific ferroptosis sensitivity	Early-stage R&D	131686669

MCT, monocarboxylate transporter; HIF, hypoxia-inducible factor; VEGF, vascular endothelial growth factor; LDHA, lactate dehydrogenase A; ANGPT, angiopoietin; RCC, renal cell carcinoma; SREBP, sterol regulatory element-binding protein; TREM2, triggering receptor expressed on myeloid cells - 2; FASN, fatty acid synthase; TFR1, transferrin receptor 1; DMT1, divalent metal transporter 1; FPN, ferroportin; OxPLS, oxidized phospholipids; TME, tumor microenvironment.

**Table 2:** Experimental platforms and pharmacodynamic readouts to operationalize metabolism-informed TAM re-education.

Toolkit	Platform / biomarker	What it measures	Strengths	Limits	Best use cases	Ref.
Preclinical model systems	Organoids with immune co-cultures	Tumor-intrinsic metabolism and immune interactions under controlled pH/O2/metabolites; TAM/T-cell crosstalk	Patient-specific; perturbation-ready; scalable; integrates with imaging/spatial/multiplex	Simplified stroma/vasculature; partial myeloid fidelity; metabolic drift over time	Titrate lactate/adenosine/lipid interventions; shortlist combinations; mechanism discovery	136
	Humanized xenograft models	*In-vivo* human tumor + human immune system; vascular and pharmacologic context	Human-specific targets; drug PK/PD; vessel effects (VEGF/HIF)	Incomplete myeloid reconstitution; interspecies metabolism; GVHD risk	Test ICI + metabolic agents (CD73/A2A, MCT1, HIF-2α)	137
	*Ex vivo* tumor slices	Native architecture & gradients; short-term functional PD	Patient-proximal; preserves spatial niches; rapid turnaround	Limited tissue; short culture window; throughput constraints	Bridge PK/PD to biology; visualize TAM phagocytosis/raft disruption	139
Readouts	Metabolic flux analysis	Real-time carbon/nitrogen flux through glycolysis, TCA, FAO	Mechanistic resolution; quantifies pathway engagement	Specialized analytics; tissue requirement	Confirm on-target effect of LDH/MCT/FASN modulation	140 142
	Single-cell spatial multi-omics	Cell states & niches (myeloid/TAM programs; NT5E/ADORA2A; PLIN/HILPDA)	Topographic context; heterogeneity; discovery-ready	Cost/complexity; standardization needs	Patient selection & pharmacodynamic mapping; mechanism of resistance	143
	Metabolic imaging (HP-13C MRI; PET tracers)	Live flux (k_PL_), hypoxia, glutamine uptake	Noninvasive; whole-tumor; longitudinal	Access & sensitivity vary; lesion heterogeneity	Early PD anchor; adaptive enrichment	146 147 148
	Functional assays (phagocytosis/ADCP)	Macrophage effector function (uptake/clearance)	Actionable, mechanistic	Assay variability; *ex-vivo* dependence	Go/No-Go gating for macrophage-centric combos	150
Biomarker	Plasma ANGPT2 (RCC)	Myeloid-angiogenesis coupling; vascular tone	Accessible; links to ICI-TKI biology	Inflammation confounders; needs context imaging	Monitor RCC combos; enrich ANGPT2-high cases	153
	HP-¹³C-pyruvate MRI k_PL_ (PCa & BCa)	Pyruvate→lactate flux (glycolytic pressure)	Early, noninvasive PD; spatially comprehensive	Availability; not all lesions glycolysis-dominant	Track lactate-pathway inhibition; prime-then-ICI designs	154
	Myeloid-derived IL-23 (CRPC)	AR-independent resistance circuit from myeloid cells	Biology-anchored; tissue/serum measurable	Specificity limits in systemic inflammation	CRPC enrichment; monitor anti-myeloid strategies	157
	PLIN2 (RCC)	HIF-centric lipid-droplet program; urinary detection	Noninvasive screen/follow-up; histologic concordance	Predictive value for combos under study	RCC detection/monitoring; pair with imaging	158 159

PK/PD, pharmacokinetics/ pharmacodynamics; VEGF, vascular endothelial growth factor; HIF, hypoxia-inducible factor; GVHD, graft-versus-host disease; ICI, immune checkpoint inhibitor; MCT, monocarboxylate transporter; LDH, lactate dehydrogenase; FASN, fatty acid synthase; TCA, tricarboxylic acid cycle; FAO, fatty acid oxidation; MRI, magnetic resonance imaging; ANGPT2, angiopoietin 2; RCC, renal cell carcinoma; PCA, prostate cancer; BCA, bladder cancer; CRPC, castration-resistant prostate cancer; PLIN, Perilipin.
